# Comment on Tekin et al. Novel Conservative Therapies in Migraine Management: The Impact of Fascia Exercises in a Randomized Controlled Trial. *J. Clin. Med.* 2025, *14*, 539

**DOI:** 10.3390/jcm15124657

**Published:** 2026-06-16

**Authors:** Christian Lunghi, Francesca Baroni

**Affiliations:** 1 osteoMANIa: Italian Osteopathic Practice and Research Working Group, BMS Formation, 00146 Rome, Italy; francesca@bms-formation.com; 2 BMS Formation, 75116 Paris, France

**Keywords:** musculoskeletal physiological phenomena, movement, motion, manipulation, osteopathic, exercise movement techniques, exercise therapy, mind–body therapies, bodyworks

We read with great interest the recent article by Tekin et al. (2025) [1] and sincerely appreciate the authors’ work in exploring innovative conservative therapies for migraine. We were particularly pleased to see the inclusion of an osteopathically inspired approach within an interprofessional intervention. Specifically, the study employed Fascial Pattern Exercises, performed in various positions targeting appendicular, axial, meningeal, and visceral fascia, with repeated movements and archetypal postures at the beginning and end of sessions. We welcome the inclusion of this approach and would like to emphasize its osteopathic foundations in the context of engaging the patient in participatory approaches integrated with individualized osteopathic manipulative treatment, guided by the assessment of somatic dysfunction [2]. The method was first described in an osteopathic textbook [3], which emphasizes that its correct application requires specific osteopathic skills, particularly the palpatory assessment of somatic dysfunction and related fascial patterns according to the fascial compensatory model described by Zink [4], while also incorporating Archetypal Postures and Erectorcises [5]. Representative figures illustrating the Fascial Pattern Exercises as part of the Patient Active-participative Osteopathic Approaches are provided in the Supplementary Material for reference (Figure S1: Fascial Pattern Exercises, Archetypal Postures and Erectorcises: An Example of Patient Active-Participative Osteopathic Approaches.). Over the years, our group and co-authors have refined the approach, detailing its historical roots, clinical applications, and interprofessional strategies [6,7] to promote patient biobehavioral synchronization and provide readers with full context regarding its osteopathic basis [8]. We thank the authors for integrating Patient Active-participative Osteopathic Approaches [6], and, in particular, Fascial Pattern Exercises, as part of Functional Neuromyofascial Activity [7] into their study. While we appreciate that Tekin et al. cite aspects of the osteopathic and multidisciplinary literature [9], several of these specific methodological components do not appear to be fully contextualized within their original conceptual or clinical framework. We believe that a clearer acknowledgment of these previously published contributions [2,3,4,5,6,7,8] would strengthen the scientific transparency and reproducibility of the intervention, particularly given the complexity of multimodal and integrative therapeutic strategies. Precise attribution is essential not only for recognizing prior work but also for ensuring that such approaches are correctly interpreted, implemented, and evaluated within both clinical and research settings. Therefore, we respectfully invite the authors to clarify the relationship between their intervention and the existing osteopathic literature from which these methods are evidently derived. Such clarification would be of significant value to readers and would further support the rigorous integration of osteopathic principles into interdisciplinary care models. We acknowledge the authors’ contribution to the field and anticipate that continued dialogue will foster greater methodological transparency and scientific rigor in future research, thereby supporting the integration of osteopathy, physical therapy, and behavioral interventions among healthcare professions within a multidisciplinary approach to pain management [10].

## Data Availability

The original contributions presented in this study are included in the Supplementary Material. Further inquiries can be directed to the corresponding author.

## Supplementary Materials

The following supporting information can be downloaded at: https://www.mdpi.com/article/10.3390/jcm15124657/s1, Figure S1: Fascial Pattern Exercises, Archetypal Postures and Erectorcises: An Example of Patient Active-Participative Osteopathic Approaches.
