# Supplementary material for: Comment on Tekin et al. Novel Conservative Therapies in Migraine Management: The Impact of Fascia Exercises in a Randomized Controlled Trial. J. Clin. Med. 2025, 14, 539
Source: J Clin Med. 2026 Jun 16;15(12):4657. doi: 10.3390/jcm15124657 (PMC13301721; doi:10.3390/jcm15124657)
Supplement: Supplementary file 1 [file jcm-15-04657-s001.zip › jcm-4318025-supplementary.pdf]

Journal of
Clinical Medicine

Supplementary material. Fascial Pattern Exercises, Archetypal Postures and Erectorcises: An Example of Patient Active-Participative Osteopathic Approaches.

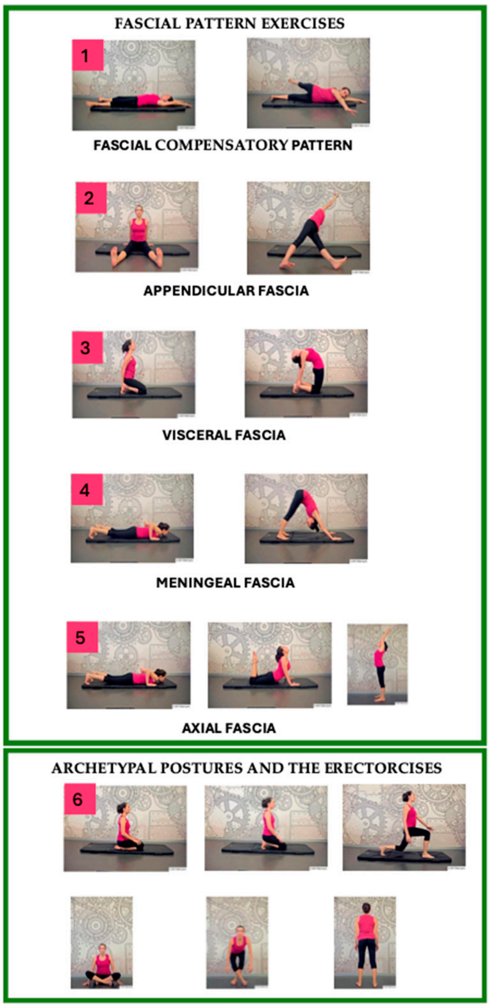

Figure S1: Fascial Pattern Exercises, Archetypal Postures and Erectorcises: An Example of Patient Active-Participative Osteopathic Approaches. Images adapted from Lunghi, C.; Baroni, F.; Alò, M. *Ragionamento clinico osteopatico*, EDRA Edizioni, Milano, 2017; pp. 186–198. Patient Active-Participatory Osteopathic Approaches (PAOA) aim to foster self-care and an internal locus of health by combining osteopathic principles with active patient engagement. These approaches integrate structural, functional, emotional, and psychological dimensions, encouraging patients to participate actively in their healing through movement, guided exercises, and lifestyle strategies. PAOA engages patients through both passive and active techniques, including functional neuromyofascial activities (FNA), fascial pattern exercises (FPE), and Archetypal Postures and Erectorcises (APE), which promote skeletal and fascial tuning, coordinated movement, and body awareness. FNA and APE can include screening of motor abilities, physical capacity, and injury risk, and can be performed in clinical sessions as assisted fascial pattern exercises to guide adaptation and monitor patient response. In this context, the osteopath provides interoceptive and proprioceptive cues while applying manipulative techniques. When functional motor abilities are assessed together with the osteopath, FNA can subsequently be practiced independently at home as brief motor breaks, using FPE and APE to support energy maintenance, mobility, and self-management. FNA, with FPE and APE, are performed in sequential phases: first, functional movement to achieve postural stabilization; second, auxiliary fascia-oriented movements, including fascial stretching, rebound elasticity, and sensory refinement through intentional breathing and body scanning; third, transitioning through functional movement or returning to a neutral posture. These participatory approaches facilitate whole-body self-tuning by aligning musculoskeletal structures and reducing dysfunction risk. Performing FNA, with FPE and APE for 3–15 minutes promotes connective tissue remodeling, tissue gliding, restoration of relationships among muscular compartments, and resetting of baseline myofascial tone. Prolonged, comfortable postures optimize biomechanical tuning and minimize fascial tone discordance. The approach emphasizes interprofessional

integration, combining osteopathic manipulative treatment, mindfulness, developmental principles, and lifestyle guidance to optimize musculoskeletal, neuromotor, and psychological function. Scoring or self-assessment tools support progress monitoring and individualized adaptation, enhancing adherence and long-term outcomes. Moreover, FNA—particularly FPE and APE—offers a transversal interprofessional strategy, fostering collaboration between osteopaths and other health or movement professionals, such as physiotherapists, occupational therapists, and instructors of body-centered disciplines like yoga, Pilates, or fascial-oriented exercise.

https://doi.org/10.3390/jcm15124657
